# Virulence Genes, Shiga Toxin Subtypes, Serogroups, and Clonal Relationship of Shiga Toxin-Producing *Escherichia Coli* Strains Isolated from Livestock and Companion Animals

**DOI:** 10.3390/ani9100733

**Published:** 2019-09-27

**Authors:** Nicolás Galarce, Beatriz Escobar, Fernando Sánchez, Esteban Paredes-Osses, Raúl Alegría-Morán, Consuelo Borie

**Affiliations:** 1 Departamento de Medicina Preventiva Animal, Facultad de Ciencias Veterinarias y Pecuarias, Universidad de Chile, 8820808 Santiago, Chile; beatrizescob@gmail.com (B.E.); fernando.sanchez@ug.uchile.cl (F.S.); ralegria@veterinaria.uchile.cl (R.A.-M.); cborie@uchile.cl (C.B.); 2 Instituto de Salud Pública de Chile, Departamento de Salud Ambiental, 7780050 Santiago, Chile; eparedes@ispch.cl; 3 Facultad de Ciencias Agropecuarias, Universidad Pedro de Valdivia, 7500908 Santiago, Chile

**Keywords:** cattle, clonality, companion animals, STEC, swine, virulence

## Abstract

**Simple Summary:**

Shiga toxin-producing *Escherichia coli* is a zoonotic pathogen that can cause severe illness in humans, and its circulating strains in the animal-human-environment interface exhibit great variability in terms of virulence and serotypes, where diverse animal species, mainly ruminants, play a fundamental role as reservoirs. Thus, the aim of this study was to characterize strains of this pathogen present in cattle, swine, dogs, and cats in the Región Metropolitana, Chile, based on virulence, serogroups, and population diversity. Based on findings, the circulating strains isolated exhibit high variability and harbor genetic determinants associated with severe illness in humans, thus highlighting that preventive and control strategies should not be focused on detecting serogroups, but instead, on detecting their determinants of virulence.

**Abstract:**

Shiga toxin-producing *Escherichia coli* (STEC) is a zoonotic pathogen that causes severe illness in humans and is an important cause of foodborne disease. In Chile, there is limited information on the virulence characteristics of this pathogen in livestock, and none in companion animals. The aim of this study was to characterize STEC strains isolated from cattle, swine, dogs, and cats, in Chile, in terms of the presence of Shiga toxin types and subtypes, virulence genes, serogroups, and clonality. One-thousand two-hundred samples were collected, isolating 54 strains (4.5%), where *stx*1a (68.5%) and *ehx*A (74.1%) were the most frequently detected virulence genes. Only one strain belonging to the most clinically relevant serogroups was identified. Pulsed field gel electrophoresis analysis showed high clonal diversity among strains isolated from cattle, while those from swine showed the same pattern. This study provides further evidence regarding cattle and swine in Chile as a potential source of a wide variety of STEC strains that could potentially cause severe illness in humans, and that companion animals do not seem to represent a relevant reservoir. It also argues that preventive and control strategies should not be focused on detecting serogroups, but instead, on detecting their determinants of virulence.

## 1. Introduction

Shiga toxin-producing *Escherichia coli* (STEC) is a zoonotic diarrheagenic pathotype of *E. coli*, whose hallmark is the ability to produce cytotoxins of the Shiga toxin family (Stx) [1]. STEC possesses more than 400 serotypes, with several that have been related to diseases in animals and humans [2]. In animals, it was described that certain STEC strains are responsible for the oedema disease of swine, diarrheal and dysenteric processes in calves and lambs, and even cases of cutaneous and renal glomerular vasculopathy in dogs [3,4]. Although their participation in different diarrheal processes in animals is recognized, their frequency is low; consequently, they are considered to be intestinal reservoirs and disseminators, with the ability to transmit the pathogen to other animals, the environment, and humans.

The intestinal carriage of STEC has been demonstrated in several animal species. In this respect, STEC presence has been reported in dairy cattle with values from 0.2% to 74% [5], and in beef cattle from 2.1% to 70.1% [6]. This high range may be due to differences in ruminal development according to age, immune response, diet, aspects of cattle management, and even climate conditions [7,8]. Additionally, STEC has been detected in sheep and goats with values ranging from 32.1% to 66.6% and from 39% to 75.3%, respectively [9,10,11], and in swine up to 35% [12]. Moreover, reports in dogs vary from 4% to 15.5%, and from 4.2% to 13.8% in cats [11,13,14].

Due to the diversity of reservoirs, routes of transmission, and low infectious dose (<100 CFU/g), infection in humans is common [2,15]. STEC strains can cause moderate to severe diarrhea, hemorrhagic colitis (HC) and hemolytic uremic syndrome (HUS) in children younger than 10 years old, and thrombotic thrombocytopenic purpura in adults and elders [16]. HUS is the most severe disease caused by STEC, and its mortality rate during the acute phase is 1%–2% [17]. In addition, HUS is the main cause of acute renal failure in children, and about 30% of these patients develop chronic kidney disease [16,18]. Although the incidence of STEC-related infections varies worldwide, its importance and impact are enormous. For example, according to official data in Chile, the incidence of HUS is 3.2/100,000 in children younger than four, with a mortality rate of 3%–5% [19,20]. One group of authors [21] conducted a meta-analysis, estimating that the global annual incidence of STEC infections in humans is 2,801,000, with 3890 cases of HUS and 230 deaths. In humans, the serotype O157:H7 is the most frequently associated with outbreaks and sporadic cases of HUS in several countries [22,23], although serogroups O26, O45, O103, O111, O121, and O145 have also been frequently associated with severe disease (therefore known as the “big six”) [23,24].

Stx are the main virulence factors of STEC, and have two types (Stx1 and Stx2), with several subtypes each. Thus, the Stx1 subfamily includes Stx1a, Stx1c, and Stx1d; while the Stx2 type includes Stx2a, Stx2b, Stx2c, Stx2d, Stx2e, Stx2f, and Stx2g [2]. Recently, the new Stx2h was described [25], which to date was only detected in a marmot species that is endemic to the Himalayas. Certain subtypes are of clinical relevance, as they are associated with more severe cases, such as HC and HUS, while others are related to uncomplicated diarrhea or are unrelated to human disease. In this respect, strains producing Stx2a, Stx2c, or Stx2d have been reported to be more virulent than those producing only Stx1 or both Stx1 and Stx2 [26]. Other virulence factors associated with STEC correspond to intimin (Eae), enterohaemolysin (EhxA), long polar fimbriae (Lpf), and STEC autoagglutinating adhesin (Saa), among others [27]. It should be noted that not all STEC strains possess the same virulence factors, constituting different virulotypes, with some being associated more frequently with severe disease in humans, such as the *stx*2/*eae*/*ehx*A virulotype [2].

Furthermore, genotypically different virulent STEC strains have emerged. For example, there are the new highly virulent O26:H11 strains harboring *stx*2 [28,29] and the enteroaggregative enterohemorrhagic *E. coli* (EAEHEC) O104:H4 [2]. The emergence of new highly virulent STEC strains challenges traditional diagnostic protocols and epidemiological studies, which are generally based on the identification of genes that were thought to be specific to the strains associated with human illness, such as *stx*1/*stx*2 and *eae*.

Determining virulence-related characteristics of circulating STEC strains may indicate the distribution of highly virulent strains in the population and therefore, enable the development of appropriate control strategies. Thus, the aim of this study was to characterize STEC strains isolated from cattle, swine, dogs, and cats in the Región Metropolitana, Chile, in terms of the presence of virulence genes, Shiga toxin subtypes, serogroups, and clonality.

## 2. Materials and Methods

### 2.1. Sample Collection

Samples were obtained through a random selection process, from intestinal content of cattle and swine (n = 300, each) at four abattoirs located in the Región Metropolitana (two abattoirs for animal species). Fecal samples (20 g approx. per animal) were obtained aseptically from rectums, in gut and tripe rooms, and collected in sterile flasks. Healthy pet dogs and cats were also randomly sampled, with prior consent from the Institutional Committee of Care and Use of Animals at the Universidad de Chile (permit code 17083-VET-UCH) and owner consent, in veterinary hospitals in the Región Metropolitana. Three hundred (300) stool samples were collected from each animal species by rectal swabbing, using sterile swabs with Cary Blair transport medium (Becton, Dickinson and Company, Franklin Lakes, NJ, USA). After collection, all samples (n = 1200) were immediately refrigerated and transported to the laboratory within four hours.

### 2.2. Sample Processing

Samples were processed according to the protocols of previous studies [13,30,31]. Briefly, feces (5 g) and swabs were enriched in 9 mL tryptone soy broth (Becton, Dickinson and Company, Franklin Lakes, NJ, USA), and each sample was homogenized and incubated overnight at 42 °C. Subsequently, 25 μL of each culture were plated onto MacConkey agar (Becton, Dickinson and Company, Franklin Lakes, NJ, USA) plates and incubated at 37 °C for 18–24 h. An aliquot from the confluent area of bacterial growth was suspended in 500 μL of sterile nuclease-free water and boiled for 15 min at 100 °C. Subsequently, the tubes were centrifuged at 26,480 g for 5 min at room temperature. Concentration and quality (260/280 absorbance ratio) of the obtained extracted DNA was measured in a nanodrop (NANO-400 micro-spectrophotometer, Hangzhou Allsheng Instruments Co., Hangzhou, China). Samples with an absorbance ratio closest to the optimal range (1.8–2.0) were kept at −20 °C for further analyses [32]. Presence of *stx*1 and/or *stx*2 genes was assessed by PCR in a LifeECO^®^ Thermocycler (Hangzhou Bioer Technology Co., Hangzhou, China) with primer sets and reaction conditions described elsewhere [33] (Table 1). A previously characterized STEC strain was used as a positive control (STEC97) [34], and *E. coli* ATCC 25922 was used as a negative control. PCR products (5 μL) were separated by electrophoresis on a 2% (wt/vol) agarose gel and visualized under LED light (GelDock, Maestrogen Inc., Hsinchu City, Taiwan) by SYBR^®^ Safe DNA Gel Stain 10,000X (Thermo-Fisher Scientific, Waltham, MA, USA). Product size was determined using Accuruler 100 bp Plus DNA ladder (Maestrogen Inc., Hsinchu City, Taiwan).

For each PCR positive sample, a maximum of 30 colonies with *E. coli* phenotype were individually plated onto MacConkey agar (Becton, Dickinson and Company, Franklin Lakes, NJ, USA) plates and subjected to the multiplex PCR described above to identify the corresponding colony harboring *stx*1 and/or *stx*2 genes. If this was not possible, isolation was repeated from the confluent growing zone. Only one isolate per sample was kept at −80 °C for further analyses.

### 2.3. Shiga Toxin Subtype Detection

Strains identified as STEC were analyzed to identify the *stx* subtype present. The detection of these subtypes was performed using the *E. coli vtx*1 and *vtx*2 Subtyping PCR Kit (Statens Serum Institut, Copenhagen, Denmark), following manufacturer instructions.

### 2.4. Virulence Gene Detection

All STEC isolates were characterized by PCR, determining the presence of several virulence genes by using PCR in a LifeECO^®^ Thermocycler (Hangzhou Bioer Technology Co., Hangzhou, China), including *ehx*A, *saa*, *eae*, *efa*1 non-O157, and *lpf*A genes (Table 1). For *ehx*A, *eae,* and *lpf*A genes, the STEC97 strain was used as a positive control. In the absence of a positive control for *efa*1 non-O157, *lpf*A, and *saa* genes, some of the PCR products obtained were sequenced. The obtained sequences were compared to the database available in GenBank^®^ (National Center for Biotechnology Information, Bethesda, MD, USA) to establish their nucleotide identity (NI), which was confirmed considering ≥97% of identity.

### 2.5. Molecular Serogrouping

Each STEC isolate was analyzed by PCR to determine if it belonged to the “big six” group, O157 and to O104 serogroups, according to protocols described in previous studies [39,40]. Table 1 shows the primers used for molecular serogrouping. Strains used as positive controls were provided by Dr. Roberto Vidal of the Instituto de Ciencias Biomédicas at the Universidad de Chile and Dr. Magaly Toro of the Instituto de Nutrición y Tecnología de los Alimentos at the Universidad de Chile.

### 2.6. Pulse-Field Gel Electrophoresis (PFGE)

This assay followed the PulseNet protocol [41]. Electrophoresis was performed using CHEF DRIII CHILLER (Bio-Rad, Hercules, CA, USA) equipment. DNA was digested with *Xba*I endonuclease, with 50U per sample (Thermo-Fisher Scientific, Waltham, MA, USA). PFGE patterns were analyzed with the GEL COMPAR II software 5.10 (Applied Maths, Sint-Martens-Latem, Belgium), using the Dice similarity coefficient with a 2% tolerance in band position [42,43,44].

### 2.7. Clustering

Results from PCR for virulence genes and Stx subtype detection, in addition to restriction patterns from PFGE (assuming that these patterns indicate clonal variability), were analyzed through the construction of a binary matrix, according to a previous study [45]. “1” was used for the presence and “0” for the absence of genes or bands from each isolate. Hierarchical clustering was performed using the similarity matrix. This was computed from the data matrix based on the report of pairwise similarities using the Jaccard coefficient, via unweighted pair group method of averages (UPGMA, determining the number of clusters using the “Elbow method”. Clustering analysis was performed using RStudio software (Integrated Development for R. RStudio, Inc., Boston, MA, USA) and the “factoextra”, “NbClust”, using RStudio software and the “ggdendro”, “ape”, and “graphics” packages. Discriminatory Power (DP) was calculated with Simpson’s Diversity Index, as reported in a previous study [46].

## 3. Results

From the 1200 samples analyzed, 4.5% (n = 54) were stx1 and/or stx2 positive. We recorded a prevalence of 17% (51/300) in cattle and 1% (3/300) in swine, while no strains were isolated from companion animals.

From the total number of strains (n = 54), 51.9% (n = 28) harbored the stx1 gen, 31.5% (n = 17) stx2, and 16.7% (n = 9) were stx1 and stx2 positive. Among other virulence genes, the most frequently detected was ehxA (74.1%, n = 40), followed by saa (70.4%, n = 38), lpfA (38.9%, n = 21), eae (1.9%, n = 1), while efa non-O157 was not detected. The Shiga toxin subtype most frequently detected was stx1a (66.7%, n = 36), followed by stx2a (29.6%, n = 16), stx2d (16.7%, n = 9), stx2c (7.4%, n = 4), stx2e (5.6%, n = 3), and stx2b (3.7%, n = 2). Subtypes stx1c, stx1d, stx2f, and stx2g were not detected.

Among the strains isolated from cattle (n = 51), most were positive for stx1 (54.9%, n = 28), followed by stx2 (27.5%, n = 14), and stx1 and stx2 (17.6%, n = 9). Regarding other virulence genes, 76.5% (n = 39) of the strains harbored the ehxA gene, 2% (n = 1) the eae gene, 68.6% (n = 35) the saa gene, and 35.3% (n = 18) the lpfA gene. In these strains, the stx subtype most frequently detected was stx1a (70.6%, n = 36), followed by stx2a (31.4%, n = 16), stx2d (17.6%, n = 9), stx2c (7.8%, n = 4), stx2b (3.9%, n = 2), and none harbored stx2e. On the other hand, all three strains isolated from swine were positive for stx2e, lpfA, and saa, and one of them also carried the ehxA gene.

Consensus sequences obtained from the sequenced amplicons showed ≥98% of NI when compared to other sequences deposited in GenBank^®^.

The detection of stx subtypes and other virulence genes showed 20 different virulotype profiles, with stx1a/ehxA/saa (29.6%, n = 16) being identified as the most frequent virulotype. Table 2 shows all the virulotypes detected according to origin.

Regarding serogroup determination, only one strain tested positive, which was an O111 strain isolated from a pig.

**Figure 1:**
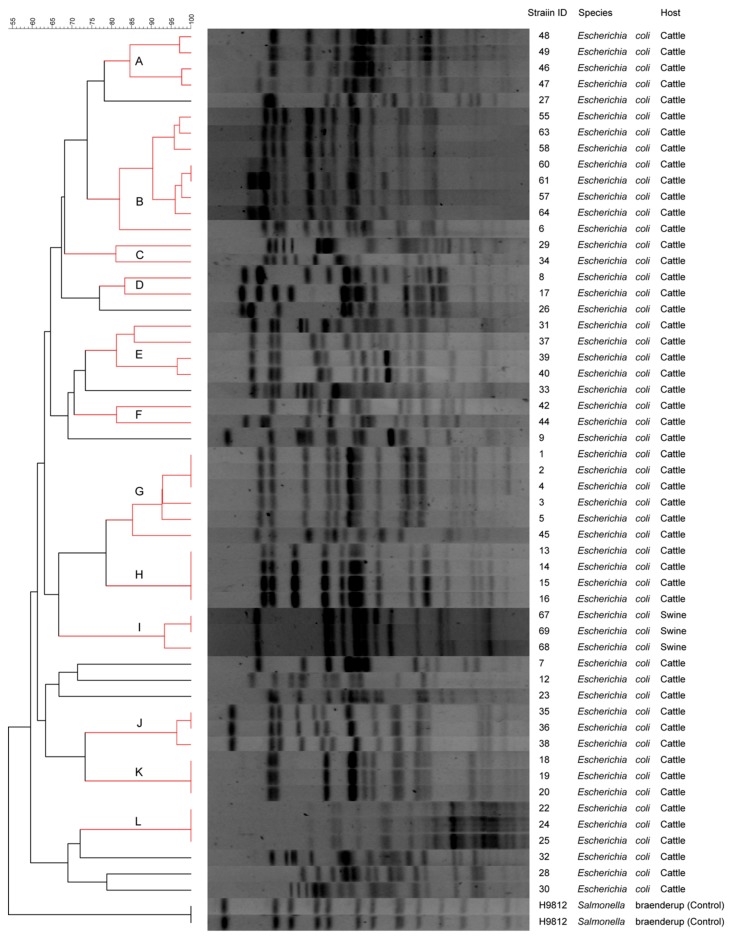
Dendrogram of 54 STEC strains isolated from cattle and swine using PFGE, showing the 12 clusters identified (A to L) in red.

**Figure 2:**
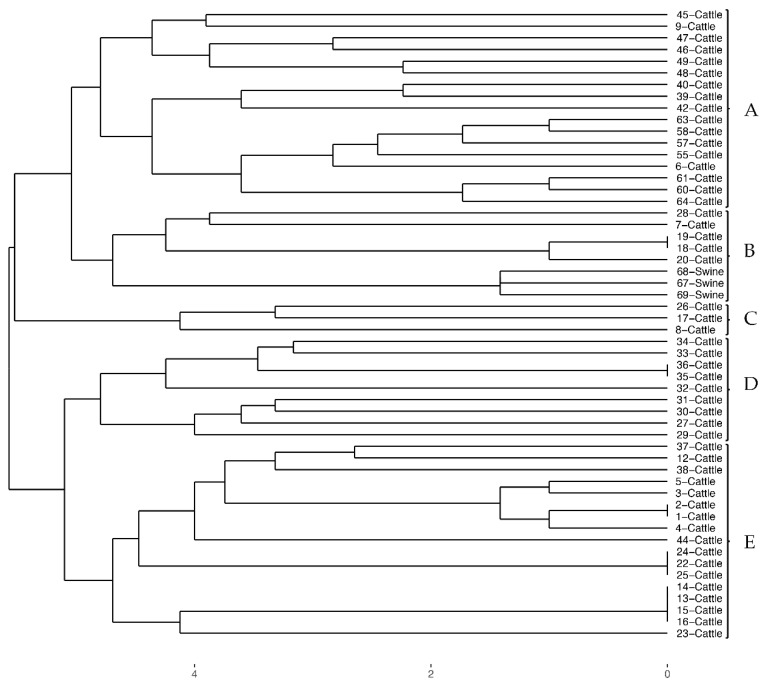
Dendrogram resulting from combined PFGE and PCR data obtained from all 54 STEC strains isolated, showing five clusters (A to E).

Through the PFGE procedure, fingerprints with 15–22 bands were obtained. This resulted in 12 major clusters (A to L), using a cut-off value of 80% similarity (Figure 1). Combined PFGE and PCR results showed a higher DP, revealing the presence of five clusters (A to E). The DP measured by Simpson’s Diversity Index was 0.763 (Figure 2).

## 4. Discussion

In Chile, a limited number of studies describe the prevalence of STEC in livestock. For example, a previous study in 1997 [34] describes the presence of STEC in the intestinal contents of cattle and swine, detecting a frequency of 28.7% and 68.3% positive samples, respectively. Another study, performed some years later [47], investigated the frequency of intestinal carriage of STEC in pigs and steers, detecting the pathogen in 13.5% of cattle samples and in 6% of swine samples. Similarly, in the present study, we detected a prevalence of 17% in cattle and 1% in swine. This reduction in prevalence rates could be due to the greater biosecurity measures currently used in animal production systems, such as the Chilean swine industry.

On the other hand, studies addressing the intestinal carriage of STEC in dogs and cats are scarce at international level, with reported prevalences varying from 2.9% to 12.3% in dogs [11,48] and from 13.8% to 23.1% in cats [11]. A previous study [13] evaluated household dogs and cats from Argentina, isolating the pathogen in 15.5% samples from dogs and 8.7% from cats, highlighting that all strains isolated from dogs harbored the *stx*2 type. In addition to STEC carriage in companion animals, close contact between these animals and their owners could facilitate the transmission of STEC strains to humans. For example, a previous study [49] isolated a STEC strain from the cat of a 2-year-old girl with vomiting and bloody diarrhea. This strain harbored both *stx*1 and *stx*2 genes. The authors conclude that the girl was probably more likely the infection source for the cat rather than vice versa, but that the girl may have been reinfected by the cat. Similarly, another study [30] isolated two different *stx*2-harboring STEC strains from a dog and a cat, which were related to their owner’s sporadic case of HUS. In the present study, it was not possible to detect the pathogen in any of the 600 samples obtained from these animal species. Nevertheless, this study is the first to address the detection of this pathogen in pets in Chile.

Regarding cattle, our results show that *stx*1 was the most frequent type (54.9%), followed by *stx*2 (27.5%), and the combination of both *stx* genes (17.6%). These results are similar to those reported in another study in Chile [34], where 56.2% of STEC strains isolated from cattle harbored the *stx*1 gene. In contrast, *stx*1+*stx*2 were the main Stx encoding genes reported in Korea (80.8%) from strains isolated from cattle [50], and *stx2* was the most frequently detected *stx* type in Argentina (52%) from strains isolated from dairy cows [51]. As mentioned before, virulence of STEC strains might be higher when possessing other virulence factors, such as intimin (encoded by *eae* gene), other adhesins (encoded by *lpf*A and *saa*, among others), and enterohaemolysin (encoded by *ehx*A gene). The *eae* gene, harbored in the locus of enterocyte effacement (LEE), encodes for intimin, a bacterial adhesin with a role in the attachment to the enterocyte, which is frequently described in highly virulent strains, such as those belonging to O157:H7 serotype [52,53]. Nevertheless, in the present study, only one strain isolated from cattle carrying this gene (2%) was identified. Similarly, a previous study [54] in Argentina reported the detection of the *eae* gene in 3% of STEC strains isolated from adult cattle. In Chile, another study [34] reported that only a small proportion of STEC strains isolated from cattle (29%) harbored this gene. In the same country, a recent study [47] did not detect the *eae* gene in any of the 102 strains isolated from analyzed cattle. In this context, other adhesins, such as Saa, may have an important role in the pathogenicity of STEC. Saa was the first of these proteins to be identified in a LEE-negative strain, and the original studies carried out in vitro showed that its expression resulted in a nearly 10-fold increase in bacterial adhesion to HEp-2 cells [53]. Here, we detected the presence of the *saa* gene in 68.6% of the isolates from cattle. Our findings are similar to those reported in Brazil in a previous study [55], where 63.2% of cattle isolates carried this gene, and to another study in Chile [47], which identified this gene in the 66% of STEC cattle strains. These results may indicate a possible increase in *saa*-harboring LEE-negative STEC strains in animals, which could have the capacity to cause severe illness in humans [56,57]. Another adhesin-encoding gene, which was detected in 35.3% of the strains isolated from cattle, was the *lpf*A gene. This gene encodes for the major fimbrial subunit protein, which is able to interact with fibronectin, laminin, and collagen IV [58]. Our results are similar to those reported by a previous study [55], where *lpf*A was detected in 20.5% of strains isolated from cattle. In regard to the detection of *ehx*A, which encodes for an enterohaemolysin and is frequently detected in human STEC isolates related to mild to severe illness [59,60], in the present study, it was detected in 76.5% of the isolates from cattle. This is similar to another study in Argentina [51], which identified this gene in 77% of STEC strains isolated from dairy cows. Regarding *stx* gene subtype detection, *stx*1a (70.6%) and *stx*2a (31.4%) were identified as the most prevalent, with these subtypes being frequently linked to serious human illness [1]. Our results are similar to those reported by a past study [61] in Japan, where *stx*1a and *stx*2a were found at values of 38.4% and 46.1%, respectively, in 176 STEC strains isolated from cattle.

In the case of the STEC strains isolated from swine, all harbored the *stx*2 type. In these strains, the *lpf*A gen was detected in 100% of strains. These results are similar to those reported previously in Poland [62], where *lpf*A was detected in 100% (n = 38) of STEC strains isolated from swine; and in the United States [63], where it was detected in 85.5% (128/150) of STEC strains of pig origin. Regarding the detection of the *saa* gene, all strains isolated harbored this gene, contrary to previous studies from Switzerland and China [64,65], where none of the STEC strains analyzed (n = 93 and n = 31, respectively) possessed that gene. The other adhesin-encoding gene addressed, *eae*, was not detected in any of these strains. Thus, this is consistent with previous reports [64,65], where this gene was not detected in any of the studied STEC strains. Additionally, we detected the presence of *ehx*A in 33.3% of STEC strains of swine origin, a rate which was higher than those described previously in China and the United States [65,66], where this gene was detected in 2.15% (n = 93) and in 7% (n = 181), respectively. It should be noted that all strains isolated from swine showed the *stx*2e subtype, which is linked to swine oedema disease, but not to severe human illness [1].

All PCR determinations performed in this study allowed us to detect that the most frequent virulotype was *stx*1a/*ehx*A/*saa* (29.6%). Remarkably, most of the strains harbored *stx*1 or *stx*1+*stx*2, *ehx*A, and *saa* genes; all of these genes are widely recognized as participating in severe human illness. Interestingly, the *ehx*A gene was detected in almost all strains associated with *lpf*A and/or *saa*. The virulence profiles detected are similar to those reported previously in Brazil [55], where most of the LEE-negative STEC strains isolated from human infections, beef and dairy cattle, harbored the *ehx*A, *lpf*A, and *saa* genes. Additionally, virulotypes not only have a role in STEC pathogenesis, but also in persistence and transmission rates. Thus, one study [67] described that strains with virulotypes harboring only *stx* are eliminated from the bovine gastrointestinal tract at higher rates than strains with virulotypes containing other virulence factors, such as *eae*.

Severe human illness has been historically linked to serogroups O26, O45, O103, O111, O121, O145, and O157 [23]. However, in recent years, other serogroups have increased their participation in severe human illness, such as O104, O113, and O178. Serotype O113:H21 was found to be prevalent in the environment, being isolated from animals, food, and also from human patients with severe disease due to STEC infection. Similarly, O178:H19 was isolated from cattle and derived food in Latin America and Europe, with most strains harboring virulence encoding genes related to severe human disease (*stx*2a, *stx*2d, *ehx*A, *saa*, among others) [68]. This points to the variability of the epidemiology of STEC infections, and that not only the most detected serogroups should be routinely screened. In Chile, according to official data, the most detected serogroups during the 2010–2016 period were O157 (55.7%) and O26 (34.9%), although other non-typeable serogroups were also reported [69]. In our study, we found only one strain belonging to the addressed serogroups, corresponding to an O111 strain isolated from a pig. The detection of O111 strains in cattle and swine feces, as well as their association with severe illness in humans, is not surprising and was previously reported [70,71,72,73]. However, O111 STEC strains isolated from humans with moderate to severe illness are mainly associated with the presence of *stx*1 type, together with *eae* and *ehx*A genes [72,73,74,75].

PFGE analysis showed a high clonal diversity among isolated strains, describing 12 clades (A to L), sharing an 80% similarity. Forty-three strains were grouped into one of these clades, with clades B and G containing the majority of the strains (7 and 6 strains, respectively). All clades contained strains isolated from cattle, except clade I, which contained all the swine strains analyzed. Strains isolated from cattle sharing the same clade corresponded to samples obtained at different sampling times, from the same abattoir but from different farms. Additionally, of the seven clusters with strains sharing 100% clonal identity, only two of them (clusters H and L) were entirely integrated by strains also sharing the same virulotype within their cluster. This could suggest the presence of clones with the same virulotype distributed in a low proportion in different farms, which could be due to husbandry practices or the fact that these animals may have had a common origin, prior to their breeding in feedlots. On the other hand, strains isolated from pigs corresponded to samples obtained at different sampling times, but from the same husbandry farm. These strains showed a high clonality (93% of homology), which is not surprising because these strains harbored almost the same genes detected here, including the *stx*2e subtype, which is found almost exclusively in pigs [3]. As expected, the *eae* harboring strain (strain 7) did not group into any of the 12 clades detected, with all of them being integrated by LEE-negative strains. The high heterogeneity in cattle isolates could be related to abattoirs receiving animals from different geographical areas, production systems, and husbandry practices, which can influence their intestinal microbiota. In a different way, Chilean swine production systems are highly technological and possess strong biosecurity measures; thus, husbandry practices tend to be very similar, favoring the dissemination of particular clones highly adapted to the environmental conditions of these facilities. Similar to our results, another study [70] investigated the clonal diversity among Chilean STEC strains isolated from patients, cattle, swine, and food by PFGE, detecting only two clinical isolates and two swine isolates with the same restriction pattern. In the same way, one group of authors [76] studied the clonal relationship among 67 clinical STEC isolates in Sweden, finding a high diversity, and only with strains from the same serogroup showing the same restriction patterns.

Clustering analysis also showed a high discriminatory power, revealing the presence of five clusters (A to E), where most of the strains were grouped in clades A and E (17 strains each). In this analysis, Simpson’s Diversity Index value was 0.763, supporting the high clonal diversity observed. Additionally, and unlike PFGE, swine strains were grouped together with some cattle strains in this analysis. Thus, even strains isolated from swine that harbored different *stx* subtypes and virulence genes, obtained through PCR, and showed distinct PFGE restriction patterns, did not have strong enough differences to cause them to differ from strains isolated from cattle.

Due to the fact that STEC is frequently present in cattle and swine feces, it is not surprising that carcasses and derived meats can become contaminated with this pathogen during slaughter [77]. One of the most frequent contamination vehicles is ground beef, followed by other meat products, which are indicated as frequently associated with outbreaks of STEC O157:H7 [78]. Thus, the presence of STEC in meat products has been widely reported, where several studies describe the virulence, serological, and clonal characteristics of the isolated strains. In this context, a previous study in Italy [79] isolated the pathogen in 2% of raw beef samples (n = 250), of which 0.8% were positive for *stx*1 and 1.2% for *stx*2. None of the STEC isolates carried the *eae* gene. In addition, *stx*1c was the most frequently detected *stx* subtype (40%), followed by *stx*2g, *stx*2c, and *stx*2a + *stx*2b + *stx*2d (20% each). None of the isolates belonged to serogroups of the “big six”, nor did they belong to O104 or O157. The isolates were analyzed by PFGE, showing five distinct restriction profiles, indicating their relatively high genetic diversity. Similarly, the presence of STEC in the entire pork meat production chain was studied in Argentina [80], where the pathogen was detected in 10.7% of the 56 meat samples analyzed. The *stx*2e subtype was detected in 50% of these strains, while none harbored *eae*, *ehx*A, or *saa*. None of the isolates belonged to serogroups of the “big six”, nor did they belong to O104 or O157. More recently, another study [81] detected the presence of STEC in 13% of the ground beef samples (n = 430) acquired in butcher shops and grocery stores in Chile. In these strains, the *stx*2 gene was the most frequently detected *stx* type (61%), followed by *stx*1 (20%), and *stx*1 + *stx*2 (19%). None of the isolates tested positive for the *eae* gene, while 37.5% harbored *hly*A. The most frequently detected virulotypes corresponded to *stx*2 (41.1%), followed by *stx*2 + *hly*A (19.6%), and *stx*1 (12.5%). None of the 56 strains tested positive for serogroup O157, nor did they test positive for the big six. Considering all of the above, the presence of STEC strains in livestock represents a risk to public health, due to the possible contamination of derived meat products with strains that could produce moderate to severe illness in people. Thus, consumers should consider the risk of consuming these products when raw or undercooked.

## 5. Conclusions

Our results show that the high diversity of STEC strains circulating in the animal element of the animal-human interface in the Región Metropolitana of Chile harbors several virulence determinants related to moderate to severe illness in humans, and therefore, represents a risk for Public Health. Thus, preventive and control strategies should not be focused on detecting serogroups or serotypes, but instead, on detecting the molecular determinants of virulence.

## Figures and Tables

**Table 1:** Oligonucleotide primer sequences for virulence and O-antigen processing genes, expected product size, and references.

Target Gene	Primers (5’-3’)	Expected Product Size (bp)	Reference
*stx*1	F: CAGTTAATGTGGTGGCGAAGG R: CACCAGACAATGTAACCGCTG	348	Cebula et al. (1995) [33]
*stx*2	F: ATCCTATTCCCGGGAGTTTACG R: GCGTCATCGTATACACAGGAGC	584	Cebula et al. (1995) [33]
*saa*	F: CGTGATGAACAGGCTATTGC R: ATGGACATGCCTGTGGCAAC	119	Paton & Paton (2002) [35]
*eae*	F: TCAATGCAGTTCCGTTATCAGTT R: GTAAAGTCCGTTACCCCAACCTG	482	Vidal et al. (2004) [36]
*efa*1 non-O157	F: ACGCTGCATACAAAAATCATCT R: TCCCTATTTCTGTCTTCTGGAGT	827	De Saint-Pierre et al. (2006) [37]
*ehx*A	F: GCATCATCAAGCGTACGTTCC R: AATGAGCCAAGCTGGTTAAGCT	534	Paton & Paton (1998) [38]
*lpf*A	F: CCTTGCGTACTGTCCGTTGA R: AGCGACCAGGGTATTGCTGT	276	Vidal et al. (2007) [31]
O26_wzx_	F: GTGTGTCTGGTTCGTATTTTTTATCTG R: CCTTATATCCCAATATAGTACCCACCC	438	Toro et al. (2013) [39]
O45_wzx_	F: GGTCGATAACTGGTATGCAATATG R: CTAGGCAGAAAGCTATCAACCAC	341	Toro et al. (2013) [39]
O103_wzx_	F: TTATACAAATGGCGTGGATTGGAG R: TGCAGACACATGAAAAGTTGATGC	385	Toro et al. (2013) [39]
O111_wzx_	F: CTTCGATGTTGCGAGGAATAATTC R: GTGAGACGCCACCAGTTAATTGAAG	362	Toro et al. (2013) [39]
O121_wzx_	F: AGTGGGGAAGGGCGTTACTTATC R: CAATGAGTGCAGGCAAAATGGAG	366	Toro et al. (2013) [39]
O145_wzx_	F: CCTGTCTTTGCTTCAGCCCTTT R: CTGTGCGCGAACCACTGCTAAT	392	Toro et al. (2013) [39]
O157_wzx_	F: TCGTTCTGAATTGGTGTTGCTCA R: CTGGTGTCGGAAAGAAATCGTTC	278	Toro et al. (2013) [39]
O104_wzx_	F: TGTCGCGCAAAGAATTTCAAC R: AAAATCCTTTAAACTATACGCCC	100	Bugarel et al. (2010) [40]

F: forward; R: reverse.

**Table 2:** Virulotypes detected in STEC strains isolated from cattle and swine.

Virulotype Profile	Number of Strains (%)			Strain ID
	Cattle (n = 51)	Swine (n = 3)	Total (n = 54)	
*stx*1a/*ehx*A/*saa*/*lpf*A	3 (5.9%)	0	3 (5.6%)	1, 2, 5
*stx*1a/*ehx*A/*saa*	16 (31.4%)	0	16 (29.6%)	3, 4, 12, 13, 14, 15, 16, 32, 33, 34, 35, 36, 37, 38, 44, 48
*stx*2a/*ehx*A/*saa*	3 (5.9%)	0	3 (5.6%)	6, 55, 58
*stx*2a/*eae*/*ehx*A	1 (2.0%)	0	1 (1.9%)	7
*stx*2b/*lpf*A	2 (3.9%)	0	2 (3.7%)	8, 26
*stx*1a/*stx*2a/*ehx*A/*saa*	3 (5.9%)	0	3 (5.6%)	9, 61, 64
*stx*2c/*stx*2d	3 (5.9%)	0	3 (5.6%)	17, 18, 19
*stx*1a/*ehx*A/*lpf*A	4 (7.8%)	0	4 (7.4%)	22, 23, 24, 25
*stx*1a	4 (7.8%)	0	4 (7.4%)	28, 29, 30, 31
*stx*2a/*ehx*A/*saa*/*lpf*A	3 (5.9%)	0	3 (5.6%)	39, 40, 63
*stx*2a/*stx*2d/*saa*/*lpf*A	1 (2.0%)	0	1 (1.9%)	42
*stx*1a/*stx*2d/*ehx*A/*saa*/*lpf*A	1 (2.0%)	0	1 (1.9%)	45
*stx*1a/*stx*2a/*stx*2d/*ehx*A/*saa*/*lpf*A	2 (3.9%)	0	2 (3.7%)	46, 49
*stx*1a/*stx*2a/*stx*2d/*ehx*A/*saa*	1 (2.0%)	0	1 (1.9%)	47
*stx*1a/*lpf*A	1 (2.0%)	0	1 (1.9%)	27
*stx*2c*/stx*2d*/ehx*A	1 (2.0%)	0	1 (1.9%)	20
*stx*1a/*stx*2a/*ehx*A/*saa*/*lpf*A	1 (2.0%)	0	1 (1.9%)	57
*stx*1a/*stx*2a/*saa*	1 (2.0%)	0	1 (1.9%)	60
*stx*2e/*saa*/*lpf*A	0	2 (66.7%)	2 (3.7%)	67, 68
*stx*2e/*ehx*A/*saa*/*lpf*A	0	1 (33.3%)	1 (1.9%)	69
